# Association between serum insulin-like growth factor-1 and bone mineral density in patients with type 2 diabetes

**DOI:** 10.3389/fendo.2024.1457050

**Published:** 2024-09-27

**Authors:** Qianqian Zhao, Youqian Li, Qiuping Zhang, Mei Zhang, Bo Ban

**Affiliations:** ^1^ Department of Endocrinology, Affiliated Hospital of Jining Medical University, Jining Medical University, Jining, Shandong, China; ^2^ Department of Cardiovasology, Affiliated Hospital of Jining Medical University, Jining Medical University, Jining, Shandong, China

**Keywords:** type 2 diabetes mellitus, nonlinear, bone mineral density, cross-sectional study, insulin-like growth factor-1

## Abstract

**Background:**

Secondary osteoporosis is associated with type 2 diabetes mellitus (T2DM), and there is conflicting evidence regarding the relationship between insulin-like growth factor-1 (IGF-1) and bone mineral density (BMD) in different populations. The objective of this study was to investigate the relationship between serum IGF-1 levels and BMD in patients with T2DM.

**Method:**

A retrospective cross-sectional study was performed on a cohort of 363 patients with T2DM, comprising men aged over 50 and women who are postmenopausal. Those with no significant medical history or medication affecting BMD or IGF-1 were considered. Data analyzed included IGF-1 levels, markers of bone metabolism, and measurements of BMD. To account for age and gender variations, we calculated IGF-1 standard deviation scores (IGF-1 SDS) for further investigation.

**Results:**

A significant increase in BMD at lumbar spine (LS), femoral neck (FN), and total hip (TH) was observed as IGF-1 SDS tertiles rose. We revealed a nonlinear correlation between IGF-1 SDS and BMD at these sites, with a common inflection point identified at an IGF-1 SDS level of -1.68. Additionally, our multivariate piecewise linear regression analysis highlighted a positive association between IGF-1 SDS and BMD at LS, FN, and TH when IGF-1 SDS exceeded the inflection point (β 0.02, 95% CI 0.01, 0.04 for LS; β 0.02, 95% CI 0.01, 0.03 for FN; β 0.02, 95% CI 0.01, 0.03 for TH). Conversely, below the inflection point, this association was not significant (β -0.04, 95% CI -0.10, 0.01 for LS; β -0.04, 95% CI -0.08, 0.01 for FN; β -0.03, 95% CI -0.08, 0.01 for TH).

**Conclusion:**

These findings reveal a nonlinear relationship between IGF-1 SDS and BMD in T2DM patients. Higher serum IGF-1 levels were connected to increased bone density only after surpassing a certain threshold.

## Introduction

Osteoporosis is a metabolic bone disease characterized by reduced bone mass, impaired bone tissue microstructure, and decreased bone quality, which ultimately leads to increased bone fragility and fracture risk (1). Type 2 diabetes mellitus (T2DM) has been established as a key risk factor for secondary osteoporosis, with a significantly higher prevalence of osteoporosis among diabetic patients. Moreover, these patients face a 2 to 3-fold increased risk of osteoporotic fractures (2). Notably, approximately one-third of T2DM patients are diagnosed with osteoporosis (3, 4). Although there is no consensus on the best method for assessing fracture risk in T2DM patients, bone mineral density (BMD) remains the most commonly used assessment indicator (5). The mechanisms by which T2DM induces osteoporosis are complex and multifactorial. It is known that insulin-like growth factor-1 (IGF-1) can activate bone remodeling and has anabolic effects on bone tissue. IGF-1 is a multifaceted growth factor that exerts a significant influence on cellular processes, including proliferation, differentiation, and metabolism (6). Within the skeletal system, IGF-1 is recognized for its essential role as a regulator of osteoblastic bone formation and osteoclastic bone resorption (7). Its dynamic interplay with bone cells is paramount in maintaining bone mass and integrity, thereby ensuring the structural and functional health of the skeletal system (8). Increasing evidence highlights the close relationship between IGF-1 and BMD, underscoring the complex role of this growth factor in bone health (9, 10). In patients with T2DM, the levels of IGF-1 may be influenced by insulin resistance and hyperglycemia, leading to a diminished anabolic effect on bone, which could potentially be one of the mechanisms underlying the increased prevalence of osteoporosis in these individuals (11). Prior evidence also suggests that among postmenopausal women with T2DM, low levels of IGF-1 are associated with an elevated risk of spinal fractures (12).

However, previous studies examining the relationship between IGF-1 levels and BMD in different populations have yielded varying results. Some studies have found a positive correlation between IGF-1 and BMD (13, 14), while others have found no such association (15). Additionally, two large cohort studies observed correlations only in women (16, 17), whereas another study observed correlations only in men (18). Importantly, previous research has mainly focused on the simple correlation between IGF-1 and BMD, without addressing whether there is a threshold effect in their relationship. Therefore, the aim of this study was to clarify the relationship between IGF-1 and BMD in local T2DM patients and to explore any potential threshold effects in this relationship.

## Subjects and methods

### Study patients

This cross-sectional study, conducted from June 2021 to April 2022, enrolled 363 men and postmenopausal women aged over 50 from the Affiliated Hospital of Jining Medical University. Serum IGF-1 levels, bone turnover markers, lumbar spine (LS), femoral neck (FN), and total hip (TH) bone density data were collected for all participants, along with other laboratory tests and anthropometric measurements. All patients diagnosed with T2DM according to the World Health Organization (WHO) standards were included in the study. Exclusion criteria were applied to patients who were under 50 years of age, premenopausal women, individuals without anthropometric measurements, those without IGF-1 data, lacking bone density examinations, individuals with malignancies, severe liver, kidney, or heart diseases, or metabolic disorders involving the pituitary, thyroid, or adrenal glands. Additionally, patients undergoing blood dialysis, immunosuppressive therapy, or taking medications affecting bone metabolism (such as vitamin D and calcium) were also excluded from the study.

### Clinical and anthropometric information

Clinical characteristics include various demographic and health-related data, such as gender, age, weight, height, diabetes duration, and a thorough medical history covering conditions like hypertension, hyperlipidemia, stroke, heart disease, and kidney issues. Additionally, a patient’s medication history is also taken into account. All this data is meticulously sourced from the hospital’s electronic medical records.

Height and weight measurements are taken with a precision of 0.1 cm and 0.1 kg, respectively. The duration of T2DM is calculated from the year of diagnosis recorded in the patient’s medical records to the year of blood markers and bone density testing. This detailed approach ensures a thorough understanding of the patient’s health status, which is crucial for personalized and effective clinical management.

### Biochemical measurements

The concentration of IGF-1 was assessed using a chemiluminescent immunoassay (DPC IMMULITE 1,000 analyzer, Siemens, Germany). Fasting plasma glucose (FPG), total cholesterol (TC), triglyceride (TG), serum calcium, and serum phosphorus levels were determined using an automated biochemical analyzer. Furthermore, a variety of hormones and bone metabolism markers, including parathyroid hormone (PTH), calcitonin, 25-hydroxyvitamin D, and relevant indicators, were evaluated using a luminescence immunoassay system (Cobas e602, Roche, Shanghai). Additionally, the measurements of estradiol and testosterone were performed using a fully automated chemiluminescent immunoassay analyzer (ADVIA Centaur XP, Siemens, Germany). Moreover, insulin and C-peptide levels were measured using the electrochemiluminescence method (Cobas e801, Roche, Shanghai). To ensure the reliability and applicability of our results, SDS for IGF-1 was calculated using reference values from a healthy population matched for age and gender. This approach allows for a standardized comparison of IGF-1 levels, accounting for the natural variation observed in the general population (19).

### Measurement of BMD

Dual-energy X-ray absorptiometry (DEXA) is a widely accepted diagnostic tool to quantify BMD in grams per square centimeter (g/cm²). This non-invasive imaging method offers precise and dependable assessments of bone density, crucial for detecting and monitoring bone health conditions. Our study involved each participant undergoing a comprehensive DEXA scan focusing on three key regions: LS, FN, and TH. These areas are particularly pertinent as they are commonly affected by osteoporosis and other bone-related ailments.

According to the guideline (20), the classification of bone health is as follows: Bone density is considered normal when the T-score is -1.0 or higher. Osteopenia, indicating reduced bone density without osteoporosis, falls within a T-score range of -1.0 to -2.5. Osteoporosis is diagnosed when the T-score falls below -2.5, indicating a significantly heightened risk of fractures and related complications. Adhering to these standardized criteria ensures a consistent and accurate assessment of bone health across our patient population.

### Statistical analysis

Continuous variables are presented as either mean ± standard deviation or median (interquartile range), with categorical variables reported as counts and percentages. Smooth curve fitting is performed to identify any potential non-linear associations between IGF-1 SDS and BMD. Drawing upon the smoothly rendered curve visualization, an enhanced evaluation of the threshold correlation between IGF-1 SDS and BMD is subsequently performed through the application of a segmented multiple linear regression analysis. In all analyses, P<0.05 is considered statistically significant. Statistical analysis is conducted using R 4.0.2.

## Results

### Participant characteristics

Among the 363 participants, comprising 190 males and 173 females, there were 128 individuals with reduced BMD, 30 with osteoporosis, and 205 with normal bone mass. Of the patients, 63.91% were treated with biguanides, 44.63% with sulfonylureas, and 38.02% with insulin. Only 3.86% were on thiazolidinediones, and 3.03% received dipeptidyl peptidase-4 (DPP-4) inhibitors. The mean FPG level was 6.92 mmol/L, ranging from 5.33 to 9.15 mmol/L. The average HbA1c was 8.86%, with a standard deviation of 2.06%. IGF-1 levels were measured, with an average of 139.00 ng/mL (interquartile range from 109.00 to 169.50 ng/mL). The IGF-1 SDS, a measure of the individual’s IGF-1 level in relation to the age- and sex-matched reference population, had an average of 0.33 (ranging from -0.57 to 1.25). The average BMD for the LS, FN, and TH were 1.07 g/cm², 0.90 g/cm², and 0.95 g/cm², respectively (**Table 1**).

**Table 1:** Clinical characteristics of the study population.

Variables	All
Participants	363
Age (years)	60.8 ± 6.9
Gender (Male)	190 (52.34%)
Height (cm)	165.05 ± 8.09
Weight (kg)	70.00 (61.00-80.00)
BMI (kg/m^2^)	25.60 ± 3.92
Duration (years)	10.50 (5.00-17.00)
FPG (mmol/L)	6.92 (5.33-9.15)
HbA1c (%)	8.86 ± 2.06
Fasting insulin (mIU/l)	7.78 (5.07-15.20)
Fasting C-peptide (ng/ml)	1.73 (1.02-2.46)
25-(OH)D (ng/mL)	16.25 (12.51-21.30)
Osteocalcin (ng/mL)	11.45 (9.13-14.44)
P1NP (pg/mL)	38.39 (31.17-49.93)
β-CTX (pg/mL)	307.15 (117.08-482.43)
Ca (mmol/L)	2.27 ± 0.11
P (mmol/L)	1.25 ± 0.20
IGF-1 (ng/mL)	139.00 (109.00-169.50)
IGF-1 SDS	0.33 (-0.57-1.25)
TG (mmol/L)	1.23 (0.87-1.87)
TC (mmol/L)	4.32 (3.57-5.02)
LS BMD (g/cm^2^)	1.07 ± 0.18
LS T-score	-0.20 (-1.10-1.20)
FN BMD (g/cm^2^)	0.90 ± 0.15
FN T-score	-0.35 (-1.52-0.45)
TH BMD (g/cm^2^)	0.95 ± 0.16
TH T-score	-0.30 (-1.12-0.83)
Estradiol (pg/mL)	26.10 (15.13-37.71)
Testosterone (ng/mL)	1.56 (0.15-3.76)
Diabetes treatment (n, %)	
Sulfonylurea	162 (44.63%)
Glinide	17 (4.68%)
Biguanide	232 (63.91%)
Thiazolidinedione	14 (3.86%)
Alpha glucosidase inhibitor	145 (39.94%)
DPP-4 inhibitors	11 (3.03%)
SGTL2 inhibitors	52 (14.33%)
GLP-1 agonists	4 (1.10%)
Insulin	138 (38.02%)
Diagnosis	
Normal bone mass	205 (56.47%)
Osteopenia	128 (35.26%)
Osteoporosis	30 (8.26%)

BMI, body mass index; FPG, fasting plasma glucose; HbAlc, hemoglobin Alc; 25-(OH)D, 25-hydroxyvitamin D; PINP, procollagen type 1 N-terminal propeptide; B-CTX, B-C-terminal telopeptide of type 1 collagen; Ca, calcium; P, phosphorus; IGF-I SDS, Insulin-like growth factor-1 standard deviation score; TG, triglyceride; TC, total cholesterol; LSBMD, lumbar spine bone mineral density; FNBMD, femoral neck bone mineral density; THBMD, total hip bone mineral density; DPP-4, dipeptidyl peptidase-4; SGT2, sodium-glucose transporter 2; GLP-1, glucagon-like peptide-1.

After stratifying the participants into tertiles according to their IGF-1 SDS, no notable variances were noted in a range of factors, encompassing age, weight, BMI, FPG, fasting insulin, fasting C-peptide, 25-hydroxyvitamin D levels, osteocalcin, P1NP, β-CTX, calcium, phosphorus, TG, and TC. However, a notable trend was observed in the average BMD across the three sites (LS, FN, TH) as the IGF-1 SDS tertile increased. Specifically, the average BMD at these sites showed a significant increase with each higher IGF-1 SDS tertile. Correspondingly, the prevalence of osteoporosis significantly decreased as individuals were grouped into higher IGF-1 SDS tertiles, as detailed in **Table 2**.

**Table 2:** The baseline characteristics of participants stratified by IGF-1 SDS.

Variables	IGF-1 SDS			P
	Tertile 1	Tertile 2	Tertile 3	
Participants	121	121	121	
Age (years)	60.25 ± 6.77	61.21 ± 6.58	60.93 ± 7.51	0.541
Gender				<0.001
Female	85 (70.25%)	55 (45.45%)	33 (27.27%)	
Male	36 (29.75%)	66 (54.55%)	88 (72.73%)	
Height (cm)	162.68 ± 7.93	165.10 ± 8.00	167.38 ± 7.72	<0.001
Weight (kg)	69.00 (60.00-75.00)	70.00 (62.00-80.00)	71.00 (65.00-80.00)	0.111
BMI (kg/m2)	25.58 ± 4.28	25.76 ± 3.83	25.45 ± 3.65	0.822
Duration (years)	10.00 (4.00-15.00)	12.00 (7.00-18.00)	10.00 (4.00-17.00)	0.299
FPG (mmol/L)	7.20 (5.46-9.05)	6.92 (5.30-8.97)	6.80 (5.26-9.40)	0.556
HbA1c (%)	9.26 ± 2.09	8.83 ± 2.16	8.48 ± 1.85	0.013
Fasting insulin (mIU/l)	7.71 (4.95-16.47)	8.50 (5.24-13.35)	6.70 (5.09-15.60)	0.890
Fasting C-peptide (ng/ml)	1.44 (0.72-2.04)	1.75 (1.10-2.59)	1.94 (1.35-2.59)	0.002
25-(OH)D (ng/mL)	15.03 (12.50-19.61)	16.70 (11.84-21.35)	17.36 (13.84-22.66)	0.129
Osteocalcin (ng/mL)	11.61 (9.30-14.43)	11.63 (9.26-14.36)	10.95 (8.96-14.04)	0.324
P1NP (pg/mL)	41.09 (31.43-50.30)	38.42 (30.29-52.26)	37.25 (31.63-48.70)	0.414
β-CTX (pg/mL)	296.90 (106.38-488.20)	299.70 (33.34-457.20)	328.05 (165.75-514.90)	0.438
Ca (mmol/L)	2.26 ± 0.09	2.27 ± 0.11	2.29 ± 0.12	0.133
P (mmol/L)	1.26 ± 0.19	1.23 ± 0.22	1.25 ± 0.18	0.506
IGF-1 (ng/mL)	99.80 (83.10-113.00)	140.00 (128.00-152.00)	186.00 (167.00-209.00)	<0.001
IGF-1 SDS	-0.98 (-1.54–0.57)	0.33 (0.06-0.65)	1.62 (1.26-2.07)	<0.001
TG (mmol/L)	1.17 (0.81-1.64)	1.30 (0.93-1.98)	1.27 (0.95-1.90)	0.050
TC (mmol/L)	4.34 (3.62-5.13)	4.49 (3.62-5.01)	4.19 (3.52-4.93)	0.843
Estradiol (pg/mL)	19.68 (11.80-34.95)	21.91 (13.43-36.13)	30.71 (23.28-38.21)	<0.001
Testosterone (ng/mL)	0.20 (0.14-2.02)	2.23 (0.16-3.81)	3.07 (0.26-4.49)	<0.001
LS BMD(g/cm2)	1.04 ± 0.19	1.07 ± 0.18	1.11 ± 0.17	0.005
FN BMD(g/cm2)	0.86 ± 0.16	0.89 ± 0.16	0.93 ± 0.14	0.001
TH BMD(g/cm2)	0.92 ± 0.16	0.94 ± 0.16	0.98 ± 0.14	0.003
Diagnosis				0.034
Normal bone mass	62 (51.24%)	63 (52.07%)	80 (66.12%)	
Osteopenia	44 (36.36%)	47 (38.84%)	37 (30.58%)	
Osteoporosis	15 (12.40%)	11 (9.09%)	4 (3.31%)	

BMI body mass index, FPG fasting plasma glucose, HbAlc: hemoglobin Alc; 25-(OH)D 25-hydroxyvitamin D: PINP, procollagen type 1 N-terminal propeptide; B-CTX, B- C-terminal telopeptide of type I collagen; Ca, calcium; P, phosphorus; IGF-1 SDS, Insulin-like growth factor-1 standard deviation score; TG, triglyceride; TC, total cholesterol; LSBMD, lumbar spine bone mineral density; FNBMD, femoral neck bone mineral density; THBMD, total hip bone mineral density.

### Assessing the correlation between IGF-1 SDS and BMD

During the univariate analysis phase, we established correlations between several clinical parameters and the BMD at the LS, FN and TH. As illustrated in **Table 3**, a significant positive association was identified between the IGF-1 SDS and the BMD at each of the specified sites (LS, FN, and TH)(P<0.001), denoting a highly statistically significant relationship. Furthermore, a positive correlation was also observed for other variables such as gender, height, weight, BMI, estradiol and testosterone with the BMD at the LS, FN, and TH (P<0.05). Additionally, the level of 25-hydroxyvitamin D showed a positive correlation specifically with the BMD at the FN and TH.

**Table 3:** Association between BMD and different variables.

Variables	LS BMD	FN BMD	TH BMD
	β 95% CI P	β 95% CI P	β 95% CI P
Age (years)	-0.01 (-0.01, -0.00) <0.001	-0.01 (-0.01, -0.01) <0.001	-0.01 (-0.01, -0.01) <0.001
Gender			
Female	reference	reference	reference
Male	0.08 (0.04, 0.12) <0.001	0.12 (0.09, 0.15) <0.001	0.11 (0.08, 0.14) <0.001
Height (cm)	0.01 (0.01, 0.01) <0.001	0.01 (0.01, 0.01) <0.001	0.01 (0.01, 0.01) <0.001
Weight (kg)	0.01 (0.01, 0.01) <0.001	0.01 (0.01, 0.01) <0.001	0.01 (0.01, 0.01) <0.001
BMI (kg/m^2^)	0.01 (0.01, 0.02) <0.001	0.01 (0.01, 0.02) <0.001	0.01 (0.01, 0.02) <0.001
Duration (years)	-0.01 (-0.01, 0.01) 0.538	-0.01 (-0.01, 0.01) 0.076	-0.01 (-0.01, 0.01) 0.114
FPG (mmol/L)	-0.01 (-0.01, 0.01) 0.237	-0.01 (-0.01, 0.01) 0.054	-0.01 (-0.01, -0.01) 0.010
HbA1c (%)	-0.01 (-0.02, -0.01) 0.018	-0.01 (-0.02, -0.01) 0.001	-0.01 (-0.02, -0.01) 0.001
Fasting insulin (mIU/l)	-0.01 (-0.01, 0.01) 0.164	-0.01 (-0.01, 0.01) 0.312	-0.01 (-0.01, 0.01) 0.351
Fasting C-peptide (ng/ml)	0.01 (-0.01, 0.01) 0.079	0.01 (-0.01, 0.01) 0.129	0.01 (-0.01, 0.01) 0.051
25-(OH)D (ng/mL)	0.01 (-0.01, 0.01) 0.570	0.01 (0.01, 0.02) 0.015	0.01 (0.01, 0.02) 0.047
Osteocalcin (ng/mL)	-0.01 (-0.01, 0.01) 0.191	-0.01 (-0.01, 0.01) 0.341	-0.01 (-0.01, 0.01) 0.086
P1NP (pg/mL)	-0.01 (-0.01, -0.02) 0.003	-0.01 (-0.01, -0.02) <0.001	-0.01 (-0.01, -0.02) <0.001
β-CTX (pg/mL)	-0.01 (-0.01, -0.02) 0.007	-0.01 (-0.01, -0.02) 0.011	-0.01 (-0.01, -0.02) 0.001
Ca (mmol/L)	0.02 (-0.16, 0.20) 0.86	-0.01 (-0.15, 0.14) 0.954	0.01 (-0.15, 0.16) 0.963
P (mmol/L)	0.09 (-0.01, 0.19) 0.071	0.07 (-0.01, 0.15) 0.082	0.08 (-0.01, 0.16) 0.073
IGF-1 SDS	0.02 (0.01, 0.03) 0.010	0.02 (0.01, 0.03) <0.001	0.02 (0.01, 0.03) 0.001
TG (mmol/L)	0.01 (-0.01, 0.01) 0.238	0.01 (-0.01, 0.01) 0.065	0.01 (-0.01, 0.01) 0.082
TC (mmol/L)	0.01 (-0.01, 0.02) 0.821	-0.01 (-0.02, 0.01) 0.293	-0.01 (-0.02, 0.01) 0.226
Estradiol (pg/mL)	0.01 (0.01, 0.01) 0.006	0.01 (0.01, 0.01) 0.035	0.01 (0.01, 0.01) 0.025
Testosterone (ng/mL)	0.02 (0.01, 0.03) <0.001	0.02 (0.01, 0.03) <0.001	0.02 (0.01, 0.02) <0.001

BMI, body mass index; FPG, fasting plasma glucose; HbAlc, hemoglobin Alc; 25-(OH)D, 25-hydroxyvitamin D; PINP, procollagen type 1 N-terminal propeptide; B-CTX P-C-terminal telopeptide of type I collagen; Ca, calcium; P, phosphorus; IGF-1 SDS, Insulin-like growth factor-1 standard deviation score; TG, triglyceride; TC, total cholesterol; LSBMD, lumbar spine bone mineral density; FNBMD, femoral neck bone mineral density; THBMD, total hip bone mineral density, P<0.05 is considered to be statistically significant.

Conversely, a negative correlation was noted between age, HbA1c, P1NP, and β-CTX with the BMD of LS, FN, and TH. This implies that as these variables increase, the BMD tends to decrease, which is particularly concerning for age and HbA1c, as they are markers of long-term health and diabetes control, respectively. However, the relationship between BMD at the LS, FN, and TH and other factors such as duration of exposure to certain conditions, osteocalcin levels, TC, TG, fasting insulin, fasting C-peptide, Ca, and P was not found to be statistically significant (P>0.05).

### Analysis of nonlinear relationships between IGF-1 SDS and BMD

**Figure 1:**
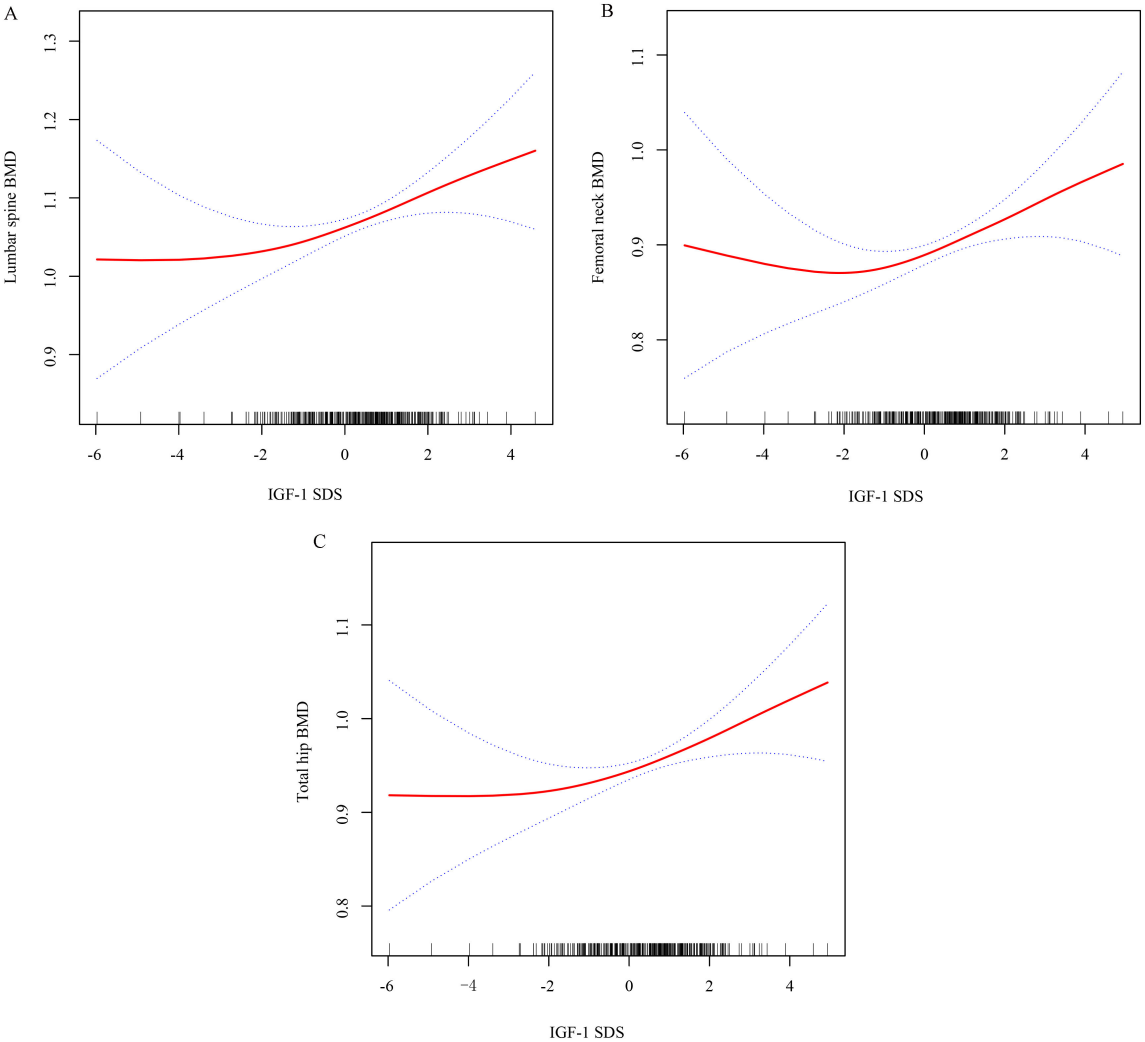
Utilizing smooth curve fitting to analyze the correlation between IGF-1 SDS and BMD at the lumbar spine **(A)**, femoral neck **(B)**, and total hip **(C)**. Adjustment variables: age, sex, diabetes treatment, BMI, FPG, HbA1c, estradiol, testosterone, 25-hydroxyvitamin D, P1NP, and β-CTX.

In this study, the analysis of nonlinear correlations between IGF-1 SDS and BMD at the LS, FN, and TH utilized smooth curve fitting. The adjustment for potential confounders such as age, sex, diabetes treatment, BMI, FPG, HbA1c, estradiol, testosterone, 25-hydroxyvitamin D, P1NP, and β-CTX revealed a nonlinear connection between IGF-1 SDS and BMD at LS, FN, and TH. The results within this investigation indicated an inflection point for each location, indicating that the variation in IGF-1 SDS and BMD for LS, FN, and TH can be categorized into two distinct phases (**Figure 1**). Furthermore, this nonlinear relationship remains consistent across both males and females (**Supplementary Figure 1**).

The findings from the simple linear regression analysis are summarized in **Table 4**, where a positive correlation was independently observed between IGF-1 SDS and BMD for LS, FN, and TH after adjusting for confounding factors (β 0.01, 95% CI 0.01, 0.03; P = 0.040 for LS; β 0.02, 95% CI 0.01, 0.03; P = 0.013 for FN; β 0.01, 95% CI 0.01, 0.03; P = 0.012 for TH). The two-part segmented linear regression analysis was performed, identifying a common inflection points for IGF-1 SDS at -1.68. Specifically, a significant positive correlation was observed between IGF-1 SDS and BMD for LS, FN, and TH when the IGF-1 SDS values exceeded the inflection point (β 0.02, 95% CI 0.01, 0.04; P = 0.004 for LS; β 0.02, 95% CI 0.01, 0.03; P < 0.001 for FN; β 0.02, 95% CI 0.01, 0.03; P = 0.001 for TH). In contrast, when the IGF-1 SDS levels were below the inflection point, no significant relationship was found between IGF-1 SDS and BMD for LS, FN, and TH (P > 0.05).

**Table 4:** Threshold effect analysis for the relationship between the IGF-1 SDS and BMD.

Models	LS BMD	FN BMD	TH BMD
	Adjusted β (95%CI) P value	Adjusted β (95%CI) P value	Adjusted β (95%CI) P value
Model I			
One line slope	0.01 (0.01, 0.03) 0.040	0.02 (0.01, 0.03) 0.013	0.01 (0.01, 0.03) 0.012
Model II			
Turning point (k)	-1.68	-1.68	-1.68
< k slope 1	-0.04 (-0.10, 0.01) 0.118	-0.04 (-0.08, 0.00) 0.078	-0.03 (-0.08, 0.01) 0.174
> k slope 2	0.02 (0.01, 0.04) 0.004	0.02 (0.01, 0.03) <0.001	0.02 (0.01, 0.03) 0.001
LRT test	0.030	0.014	0.040

Model I, linear analysis, Model II, non-linear analysis. LRT test, logarithmic likelihood ratio test. (P<0.05 means Model II is significantly different from Model I, which indicates a non-linear relationship); Adjustment variables: age, sex, diabetes treatment, BMI, FPG, HbAlc, estradiol, testosterone, 25-hydroxyvitamin D, PINP, and B-CTX P <0.05 is considered to be statistically significant.

## Discussion

This study showed a significant positive correlation between IGF-1 SDS and BMD at the among patients with T2DM. Intriguingly, we delved into the non-linear relationship between IGF-1 SDS and BMD at these sites, identifying inflection points at -1.68 for the LS and TH, and -1.64 for the FN. To be more precise, a positive association was observed between IGF-1 SDS and BMD of the LS, FN, and TH when IGF-1 SDS values exceeded these inflection points. Conversely, when IGF-1 SDS values fell below these thresholds, no significant association was found between IGF-1 SDS and BMD at the LS, FN, and TH.

IGF-1 plays a crucial role in bone metabolism, particularly in patients with diabetes, where osteoporosis is a prevalent and severe complication (2). Individuals with T2DM are often at risk for decreased bone density, making it essential to understand the relationship between IGF-1 and BMD for effective prevention and treatment of osteoporosis. However, previous studies on the relationship between IGF-1 and BMD have reported inconsistent results (13–18), with a predominant focus on linear associations and limited attention given to potential non-linear relationships.

The results of this research indicated a substantial positive relationship between IGF-1 SDS and BMD at the LS, FN, and TH in individuals diagnosed with T2DM. This discovery is consistent with a prior investigation where a sample of 391 patients, including men aged 50 and older and postmenopausal women with T2DM, underwent analysis in a retrospective, cross-sectional study. This study found a positive link between IGF-1 concentrations and BMD in this specific patient cohort (18). Additionally, a recent research effort focused on young patients with Cushing’s disease explored the connection between IGF-1 and low bone density, revealing a notable positive correlation between IGF-1 and BMD at both the LS and FN (21). Utilizing Mendelian randomization methods, researchers have shown that heightened IGF-1 levels are linked to reduced fracture risk, suggesting that increased IGF-1 concentrations might enhance BMD and overall skeletal integrity (13).

Nonetheless, conflicting data exists concerning the relationship between IGF-1 and BMD. Lloyd and colleagues reported no significant connection between IGF-1 levels and BMD at the LS or TH in their own investigation (15). In a comprehensive study involving postmenopausal women, no substantial association was observed between IGF-1 levels and BMD at critical skeletal locations like the LS and FN (22). Furthermore, an analysis of recent cross-sectional data revealed a positive correlation between IGF-1 and osteocalcin levels in T2DM patients, although no such link was found with BMD at the LS, FN, or other bone turnover markers (23). Our own research uncovers a positive association between IGF-1 SDS and BMD in patients with T2DM, underscoring the importance of regular monitoring of IGF-1 levels for early osteoporosis detection in this demographic.

Furthermore, we delved deeper into the non-linear correlation between IGF-1 and BMD at the LS, FN, and TH by utilizing smooth curve fitting techniques. It was observed that the significance of the IGF-1 and BMD relationship at these bone sites only emerges when the IGF-1 SDS concentrations surpass a specific threshold. This implies that the impact of IGF-1 on bone density is not linear, but rather demonstrates a threshold effect. This occurrence highlights the intricate regulatory function of IGF-1 in bone metabolism. Serving as a pivotal growth factor, IGF-1 plays a critical role in bone development and upkeep by enhancing the multiplication and specialization of bone cells, thereby influencing bone density (24, 25). Previous studies have also emphasized the importance of circulating IGF-1 threshold levels for achieving peak bone mass (26), and indicated that threshold concentrations of circulating IGF-1 are necessary for normal bone growth (27). In a cross-sectional, retrospective study of 391 patients, including men over 50 and postmenopausal women with type 2 diabetes, IGF-1 was positively correlated with FN and TH BMD in men. However, no significant correlation was observed between serum IGF-1 and BMD in women, likely due to their lower IGF-1 levels compared to men (18). In another study investigating the relationship between IGF-1 and BMD, the failure to observe a correlation might be attributed to the significantly lower IGF-1 concentrations observed in individuals over 60 years compared to those under 60 (28). Furthermore, in a study reporting no relationship between IGF-1 and BMD, the average IGF-1 concentration was 23 nmol/L, which may also be attributed to the low IGF-1 levels (15).

In the present study, we revealed a non-linear relationship between IGF-1 SDS and BMD and identified a key inflection point. The relationship between IGF-1 SDS and BMD was significant only when IGF-1 SDS concentrations exceeded -1.68. The main contribution of this study is that it not only confirms the positive correlation between IGF-1 and BMD but also reveals how this relationship changes at different IGF-1 levels. Compared to traditional linear models, this non-linear analysis method provides a more comprehensive reflection of IGF-1’s impact on bone density, offering significant research innovation and practical application value. Elevating serum IGF-1 concentrations could serve as a crucial clinical objective for shielding against fractures in patients with T2DM.

Several potential mechanisms may underpin the protective role of IGF-1 against BMD loss. IGF-1 plays a pivotal role in promoting proliferation of osteoblastic lineage cells, enhancing the functionality of mature osteoblasts, and serves as a fundamental driver of osteoblastic activity and bone formation processes (29). Moreover, it contributes to an upregulation of collagen synthesis and a concomitant decrease in its degradation, thereby maintaining optimal levels of the bone matrix and overall bone mass (30). Additionally, while the evidence is not yet definitive, IGF-1 might exert a protective influence on osteoclasts through mechanisms that are yet to be fully elucidated; such effects could further contribute to preserving bone integrity. Notably, elevated levels of IGF-1 have also been associated with increased muscle mass accrual and maintenance, which indirectly reduces the risk of fractures by improving skeletal stability and mechanical support (31, 32). This multifaceted action of IGF-1 underscores its significance in both bone metabolism and musculoskeletal health.

Our study has several limitations. In this retrospective analysis, we could only establish an associative relationship between IGF-1 and BMD, without being able to definitively infer causality. Future research necessitates large-scale, multicenter clinical trials designed to more rigorously investigate the causal link between IGF-1 levels and bone density. Furthermore, there is a need for additional basic research to expound upon the mechanistic actions of IGF-1 on both osteoblast and osteoclast functions, thus illuminating its specific impact on bone metabolism. Additionally, various confounding factors potentially influencing our findings were not exhaustively accounted for, such as dietary habits, intake of calcium and phosphorus micronutrients, sunlight exposure duration, and physical activity levels. Notably, although males with hypogonadism were not excluded from the study, adjustments were made for testosterone levels during analysis to enhance the accuracy of our assessment of the relationship between IGF-1 and BMD. Prospective cohort studies will be instrumental in verifying these relationships with greater precision. Lastly, IGF-1 levels were measured only once in our study, and a single measurement of IGF-1 may not serve as a reliable surrogate for chronic, long-term exposure of the skeleton to IGF-1. Future investigations will aim to monitor the longitudinal changes in IGF-1 levels and their implications on bone health over time.

## Conclusion

In conclusion, this study identified a non-linear relationship between IGF-1 SDS and BMD in T2DM patients. When IGF-1 levels reach a certain threshold, the elevation of serum IGF-1 levels is associated with an increase in BMD. The non-linear relationship between IGF-1 and BMD suggests that when assessing and intervening in bone health issues, dynamic changes in biomarkers and threshold effects should be fully considered for more precise and effective bone health management. These findings have significant clinical implications for bone health management in T2DM patients. Monitoring IGF-1 levels and ensuring they are above the identified inflection point may help prevent bone loss and reduce the risk of fractures in this population.

## Data Availability

The original contributions presented in the study are included in the article/**Supplementary Material**. Further inquiries can be directed to the corresponding author.
