# Supplementary material for: Association between serum insulin-like growth factor-1 and bone mineral density in patients with type 2 diabetes
Source: Front Endocrinol (Lausanne). 2024 Sep 27;15:1457050. doi: 10.3389/fendo.2024.1457050 (PMC11466823; doi:10.3389/fendo.2024.1457050)
Supplement: Supplementary file 1 [file DataSheet1.doc]

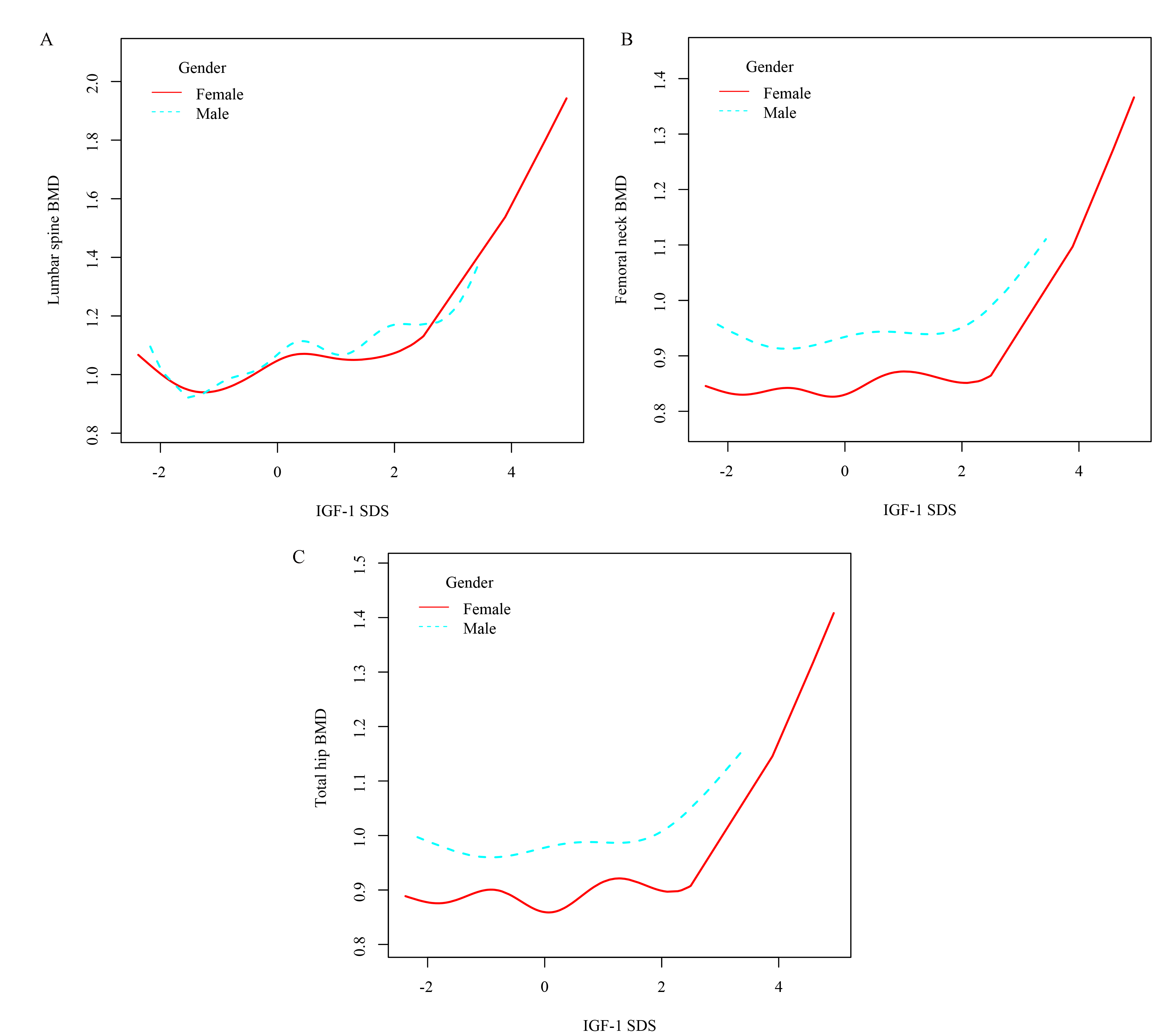


Supplementary Figure 1. Smooth curve fitting analysis of the correlation between IGF-1 SDS and BMD, stratified by gender. Adjustment variables: age, sex, diabetes treatment, BMI, FPG, HbA1c, estradiol, testosterone, 25-hydroxyvitamin D, P1NP, and β-CTX.
